# Bile Acids Elevated in Chronic Periaortitis Could Activate Farnesoid-X-Receptor to Suppress IL-6 Production by Macrophages

**DOI:** 10.3389/fimmu.2021.632864

**Published:** 2021-04-22

**Authors:** Shan Cao, Xinyu Meng, Yixuan Li, Li Sun, Lindi Jiang, Hanqing Xuan, Xiaoxiang Chen

**Affiliations:** ^1^ Department of Rheumatology, Ren Ji Hospital, Shanghai Jiao Tong University School of Medicine, Shanghai, China; ^2^ Department of Rheumatology, The First Affiliated Hospital of Wenzhou Medical University, Wenzhou, China; ^3^ Department of Rheumatology, Zhongshan Hospital, Fudan University, Shanghai, China; ^4^ Department of Urology, Ren Ji Hospital, Shanghai Jiaotong University School of Medicine, Shanghai, China

**Keywords:** bile acid, farnesoid-x-receptor, IL-6, chronic periaortitis, macrophages

## Abstract

Chronic periaortitis (CP) is a rare autoimmune disease without effective treatment. By analyzing the serum bile acid spectrum in 28 CP patients with the ultra-performance liquid chromatography-tandem mass spectrometry, we found that the bile acids were significantly altered in CP patients, with significant increases in chenodeoxycholic acid (CDCA) and glycochenodeoxycholic acid (GCDCA) and decrease in deoxycholic acid (DCA). Signaling pathway enrichment analysis from the RNA sequencing results suggested that the altered gene sets in PBMC of CP patients were associated with bile acid metabolism. Furthermore, we found that pathological concentration of CDCA could significantly inhibited IL-6 expression in RAW 264.7 cells after LPS stimulation. Since CDCA is a well-known natural high-affinity ligand for the bile acid receptor farnesoid-x-receptor (FXR) while GW4064 is the synthetic specific agonist of this receptor, we then revealed that GW4064 significantly decreased IL-6 expression in RAW 264.7 cells and bone marrow-derived macrophages but not in FXR^-/-^ macrophages upon LPS stimulation. The western blot results with the anti-FXR antibody showed significantly increased expression in the nuclear proportion, suggesting that FXR agonist promoted the transportation of FXR into the nucleus but did not increase the FXR expression in macrophages. Dual-luciferase report assay and ChIP assay demonstrated that upon activation, FXR could directly bind to the promoter site of IL-6, leading to the decreased expression of IL-6. Thus, bile acids, especially CDCA, may operate to damp inflammation *via* FXR-mediated downregulation of IL-6 in mononuclear cells and provide a protective mechanism for CP patients.

## Introduction

Bile acids are a group of water-soluble, amphipathic molecules exclusively produced in the liver. Apart from their classically known role as lipid solubilizers and their functions in managing metabolic liver disorders, bile acids are now considered to be more and more involved in maintaining systemic metabolic and immune homeostasis, and even described as potential attractive therapeutic agents (1). Increasing recognition has been raised on their nonnegligible role as important signaling molecules in the regulation of metabolically driven inflammation (1, 2) as well as autoimmune diseases. Chenodeoxycholic acid (CDCA) was reported to have a treatment role in rheumatoid arthritis as early as 1976 (3). The immunosuppressive roles of CDCA and ursodeoxycholic acid (UDCA) were also discovered in an allogeneic immune response mouse model (4). As such, Farnesoid-X-receptor (FXR), a bile acid receptor, may play a pivotal role in these signaling pathways since bile acids, especially CDCA, are well-known as natural high-affinity ligands for this nuclear hormone receptor (5).

Farnesoid-X-receptor (FXR) is a member of the orphan nuclear receptor family and is widely expressed in the liver, small intestine, adipocytes and also macrophages. Function as a bile acid activated nuclear receptor and a transcription factor, FXR can regulate the gene expression of bile acid and lipid metabolism with the activation of its natural ligand, CDCA, and participate in the process of bile acid metabolism, lipid metabolism and glucose metabolism (5). In addition, it is also noteworthy that FXR now play more of its role in the anti-inflammatory and anti-fibrosis aspects. Recent experimental and clinical evidences have indicated that, FXR agonists can significantly alleviate the damage of target organs, weaken the activation of immune cells, promote the differentiation of Tregs (6) and reduce the release of IL-1β, TNFα and other inflammatory factors, indicating that FXR plays an important anti-inflammatory role in the innate and adaptive immunity (7). FXR agonists have also achieved several good curative effects in the treatment of primary biliary cholangitis (also known as primary biliary cirrhosis), revealing the outstanding role of FXR in anti-fibrosis aspect (8, 9). Furthermore, beneficial effect of FXR agonists has also been emerged in autoimmune disease (10, 11).

Chronic periaortitis (CP) is a rare disorder and characterized by chronic idiopathic fibrosis. It is mainly manifested by inflammatory adipose tissues and collagen fibers, spreads from the adventitia of the abdominal aorta or/and the iliac artery into the retroperitoneum, wrapping around the abdominal aorta and/or iliac artery as well as inferior vena cava (12, 13). Extension of the mass could also contribute to the adhesion or obstruction of the surrounding organs. According to the extent of disease progression, CP can be divided into idiopathic retroperitoneal fibrosis (IRF), inflammatory abdominal aortic aneurysms (IAAA) and perianeurysmal retroperitoneal fibrosis (PARF), of which IRF is most common. CP was originally identified as an immune response to local atherosclerosis (14), however, emerging evidences have unveiled the fundamental of CP as an autoimmune disease. Recent associations have been found between the susceptibility of CP with HLA-DRB1 * 03 and other immune disease-related alleles (15).

Although bile acids are known to affect host metabolism and innate immunity through the activation of FXR, however, less has been described about the bile acid spectrum in CP patients and it is still unknown whether the bile acid-FXR pathway has a role in the pathogenesis or as a potential treatment for the inflammation along with fibrosis in this disease. Moreover, it was reported that the serum level of the pro-inflammatory factor IL-6 was significantly higher in active CP patients rather than healthy controls, and the IL-6 expression was dominantly elevated in CD68^+^ macrophages/monocytes (16). Since IL-6 is a very important cytokine that could be able to stimulate chronic inflammation in adipocytes and also central to pro-fibrotic interactions within fibroblasts, IL-6 may play a pivotal role contributing to the pathogenesis of CP. Therefore, we wonder if bile acid-FXR pathway could have a distinct function in the regulation of IL-6 expression in macrophages/monocytes. In our present study, we analyzed the serum bile acid spectrum of normal subjects, CP patients and disease controls. We also analyzed the transcriptomes in PBMCs of controls and CP patients and found that the pathways enriched in the analysis are highly associated with bile acid metabolism. We also demonstrated that the pathological but not physiological level of CDCA could significantly decrease the IL-6 expression in RAW264.7 after the LPS stimulation. Moreover, by using RAW 264.7 cells and bone marrow-derived macrophages (BMDMs) from wild type and FXR^-/-^ mice, we demonstrated that FXR activation by GW4064 could be able to inhibit IL-6 production. Furthermore, we showed that FXR could directly bind to the promoter region of IL-6, leading to the inhibition of IL-6 in macrophages.

## Materials and Methods

### Human Subjects

Serum samples and peripheral blood mononuclear cells of naïve CP patients without any medical treatment were collected from Department of Rheumatology, Ren Ji Hospital, Shanghai Jiao Tong University, Department of Rheumatology, Zhongshan Hospital, Fudan University and Department of Rheumatology, the First Affiliated Hospital of Wenzhou Medical University between November 2016 to July 2020, confirmed by pathology or by CT/MRI examination in line with the previous diagnostic criteria of CP (16) and exclusion of tumors and other secondary causes. Blood samples were collected after 10 h of overnight fasting. A total of 28 CP cases were finally confirmed. Meanwhile, 10 naïve Takayasu’s arteritis (TA) patients were also recruited from Department of Rheumatology, Ren Ji Hospital, Shanghai Jiao Tong University and Department of Rheumatology, Zhongshan Hospital, Fudan University. The general information, clinical symptoms and laboratory examinations of CP and TA patients were collected and documented (**Table 1**). In addition, 47 patients diagnosed with peripheral arterial disease (PAD) were recruited from the Department of Vascular Surgery, Ren Ji Hospital, Shanghai Jiao Tong University. Serums of 88 healthy controls were collected from the medical examination center of Ren Ji hospital. The study protocol was guided and approved by the Ethics Committee of Renji Hospital, and the study was performed in accordance with the principles of the Declaration of Helsinki. All participants provided written informed consent.

**Table 1:** Demographics, clinical characteristics of CP and TA patients.

Feature	Chronic Periaortitis	Takayasu’s Arteritis
**Age, years, median (IQR)**	62.5 (55.8-68)	45 (38.5-56.3)
**Gender, n (%)**		
Male	24 (85.7%)	3 (30%)
Female	4 (14.3%)	7 (70%)
**Comorbidity, n (%)**		
Sjögren’s syndrome	1 (3.6%)	0
Hypertension	4 (14.3%)	3 (30%)
Diabetes mellitus	3 (10.7%)	1 (10%)
Dyslipidemia	2 (7.1%)	2 (20%)
**Laboratory examination, median (IQR)**		
ESR (mm/h)	66 (26.0-101.0)	23 (9.3–51.5)
CRP (mg/L)	10.9 (3.4-30.2)	12.8 (3.2–66.9)
ALT (U/L)	18 (9.5-40.5)	20 (16.5-26)
γ-GT (U/L)	23 (17-92)	26.5 (20.8-117)
Total bile acid (μmol/L)	3.8 (2.3-10.6)	1.7 (0.8-3.9)
Creatinine (μmol/L)	87.5 (79-145.5)	66.2 (54.8-77.4)
IgG4 (g/L)	2.4 (0.8-3.5)	1.2(1.0-1.3)

### Examination of Serum Bile Acid

Serum samples of the patients were set at room temperature for 30 min, centrifuged at 6,000g for 10 min to obtain the serum and then stored in a refrigerator at -80°C. Ultra performance liquid chromatography-tandem mass spectrometry (UPLC-MS/MS) standard box (manufactured by Guangzhou Ke Li Mass Spectrometer Medical Instrument Co., Ltd.) was used and the examination of serum CDCA was conducted following the manufacturer’s instructions. API 3200 instrument from SCIEX Corporation was used for the examination.

### Gene Expression Profiling

Total RNA was isolated from 1 × 10^6^ peripheral blood mononuclear cells from CP or healthy controls, using the RNeasy Mini Kit (QIAGEN). RNA-seq was carried out by Genergy Biotech (Shanghai, China) and the following data analysis as well as cluster enrichment with KEGG and GSEA were then performed based on the website of Networkanalyst (17). Data were log2 transformed, followed by normalization to the 75th percentile, and corrected to the median of all samples. Features passing the quality check (variance percentile rank above 10 and read count over 5) and showing changes in expression levels equal to or more than 2-fold were selected for further analysis. A volcano plot was utilized to identify statistically significance (P < 0.05). The differentially expressed genes (DEGs) screened from the previous procedure were then put into the pathway enrichment analysis using KEGG to elucidate the biological interpretation. GSEA was also applied to enrich the pathway analysis.

### Cell Preparation

Murine macrophage cell line RAW264.7 (source from ATCC, TIB-71, male) and murine fibroblast cell line L929 (source from ATCC, CCL-1, male) were obtained from the Stem Cell Bank of the Chinese Academy of Sciences (Shanghai, China). Cells were cultured in 5% CO_2_ at 37°C and grown in DMEM medium for RAW264.7 and DMEM/F12 medium for L929 containing 10% FCS and 1% (v/v) penicillin/streptomycin (All medium and supplements were obtained from GIBCO). Bone marrow-derived macrophages were generated from total bone marrow cells flushed out from 9-14 weeks old wild type C57/B6 mice or FXR knockout (FXR^−/−^) mice and the littermates and then incubated in DMEM and GlutaMAX (Gibco, Thermo Fisher Scientific) supplemented with 10% (v/v) FCS (Gibco, Thermo Fisher Scientific), 1% (v/v) penicillin/streptomycin, and 15% (v/v) L929 conditioned medium for 7 days.

### Flow Cytometry

On Day7 of the differentiation, bone marrow-derived macrophages from FXR-/- mice and the littermates were harvested and stained with CD11b-PerCP-Cy 5.5, F4/80-APC, CD11c-FITC and CD206-PE. The cells were then measured by a BD FACSCalibur Flow Cytometer. The percentage of the differentiated macrophages (CD11b+ F4/80+), M1 macrophages (CD11b+ F4/80+ CD11c+ CD206-) and M2 macrophages (CD11b+ F4/80+ CD11c- CD206+) were then calculated. All antibodies for flow cytometry were from BD Biosciences.

### Real-Time PCR Analysis

A standard phenol-chloroform extraction was performed with Trizol reagent to isolate total RNA from RAW 264.7 cells as with bone marrow-derived macrophages from wild type and FXR^−/−^ mice and the littermates after diverse treatments. cDNA was synthesized from 1 μg of total RNA with a Reverse Transcription Kit (PrimeScript RT reagent Kit Perfect Real Time, Takara). Real-time PCR analysis was then obtained and analyzed with a SYBR label (SYBR Premix Ex Taq™, Tli RNaseH Plus, Takara) in QuantStudio™ 6 Flex Real-Time PCR System. The real-time PCR primer sequences are *Il-1β* for, GGACATGAGCACCTTCTTTTC; *Il-1β* rev, CTAATGGGAACGTCACACACC; *Tnfα* for, AAACACAAGATGCTGGGACA; *Tnfα* rev, TTGATGGTGGTGCATGAGAG; *Il-6* for, TAGTCCTTCCTACCCCAATTTCC; *Il-6* rev, TTGGTCCTTAGCCACTCCTTC; *Gapdh* for, CAGAACATCATCCCTGCATC; *Gapdh* rev, CTGCTTCACCACCTTCTTGA; *Nr1h4* for, TAGTCTTCACCACAGCCACC; *Nr1h4* rev, CAGGTTGGAATAGTAAGACGAGG. The relative count of genes was calculated by normalizing to *Gapdh* mRNA and expressed as fold change relative to the control/vehicle group.

### ELISA

The concentrations of TNFα, IL-1β and IL-6 in the relevant supernatant were performed with the Mouse TNF-alpha DuoSet ELISA Kit (DY410, R&D SYSTEMS), Mouse IL-1b DuoSet ELISA Kit (DY401, R&D SYSTEMS) and Mouse IL-6 ELISA MAX™ Kit (Cat. 431301, Biolegend) following the manufacturer’s instructions.

### Western Blot Analysis

RAW 264.7 cells with different treatments were homogenized in RIPA buffer with protease and phosphatase inhibitors. The nuclear and cytoplasmic extraction was conducted under the protocol of the Nuclear and Cytoplasmic Extraction Kit (CW0199, CWBIO, China). The protein extracts were then separated by SDS-PAGE electrophoresis and transferred to a PVDF membrane. The membrane was incubated with antibodies against Nr1h4 (sc-13063, Santa-Cruz, 1: 1000), GAPDH (Cat#2118, Cell Signaling, 1:1000) and LaminB (GTX103292, GeneTex, 1:1000) overnight at 4°C.

### Luciferase Reporter Assay

The putative FXR binding regions in the promoter of IL-6 were firstly predicted with online databases PROMO and NUBIscan. Then the regions were amplified by PCR from genomic DNA extracted from splenocytes in C57BL/6 WT mice and cloned using Kpn I and Sma I into the pGL-3 firefly reporter vector (Promega). All the constructs were verified by sequencing. Then respective luciferase reporter constructed or basic pGL-3 vector, as with Renilla plasmid and the FXR overexpression vector (Genecopoeia, Guangzhou, China) were co-transfected into 293T cells using lipofectamine 2000 (Thermo). Transfected cells were then lysed and luciferase activity was quantified with the Dual-Luciferase Reporter Assay (Promega) following the manufacturer’s instructions and normalized to the activity of the co-transfected Renilla reporter gene.

### ChIP

ChIP was performed with the EZ-Magna ChIP™ A/G Chromatin Immunoprecipitation Kit (17-10086, Millipore) following the manufacturer’s instructions. IL-6 promoter regions were amplified with the specific primer derived from the result of luciferase assay for the corresponding promoter sites by real-time PCR.

### HE and Immunohistochemical Staining

HE staining was performed by the Department of Pathology as common procedure. Anti-IL-6 polyclonal antibody (ab6672, Abcam Corporation, 1: 200) and anti-Nr1h4 polyclonal antibody (sc-13063, Santa-Cruz, 1: 50) were used to explore the expression of IL-6 and FXR in the lesion location of the disease. The bound antibodies were then visualized using EnVision reagent (K500711 kit, Dako, Denmark). Immunohistochemical results were observed by Nikon ECLIPSE Ti-s inverted microscope and analyzed by NIS-Element software. An isotype control (ab27478, Abcam, 1: 400) was used instead of each primary antibody as the negative control.

### Data Analysis

GraphPad Prism version 8.0 (GraphPad Software, San Diego, CA) was used for statistical treatment. Experimental data were shown as the mean ± SEM. Two-tailed unpaired Student’s t-test and ANOVA with multiple comparisons test were used as indicated in respective experiments.

## Result

### Bile Acids Are Altered in Chronic Periaortitis Patients

**Figure 1:**
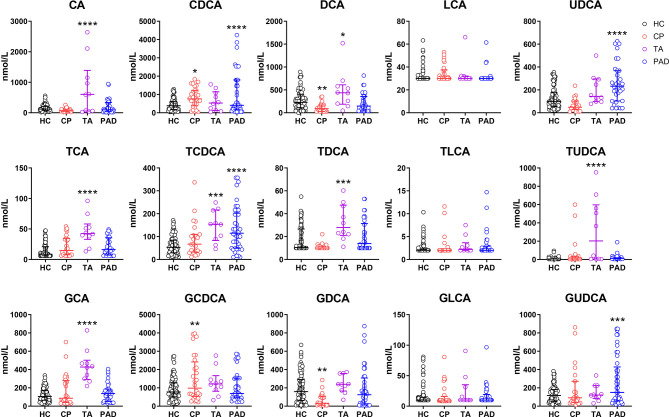
Bile Acids are Altered in Chronic Periaortitis Patients. Serum was collected from healthy controls (n=88, shown in black) as well as the patients of chronic periaortitis (CP) (n=28, shown in red), Takayasu’s arteritis (TA) (n=10, shown in purple) and peripheral artery disease (PAD) (n=47, shown in blue) and tested by ultra performance liquid chromatography-tandem mass spectrometry (UPLC-MS/MS). Bile acid spectrum in diverse groups were then analyzed. Data are shown as median (IQR). * means patients’ data comparing to healthy controls, respectively. *p < 0.05; **p < 0.01; ***p < 0.001; ****p < 0.0001, one-way ANOVA, followed by Dunnett’s multiple comparisons test. CA, cholic acid; TCA, taurocholic acid; GCA, glycocholic acid; CDCA, chenodeoxycholic acid; TCDCA, taurochenodeoxycholic acid; GCDCA, glycochenodeoxycholic acid; DCA, deoxycholic acid; TDCA, taurodeoxycholic acid; GDCA, glycodeoxycholic acid; LCA, lithocholic acid; TLCA, taurolithocholic acid; GLCA, glycolithocholic acid; UDCA, ursodeoxycholic acid; TUDCA, tauroursodeoxycholic acid; GUDCA, glycoursodeoxycholic acid.

To investigate the levels of different bile acids in CP patients, we firstly performed ultra performance liquid chromatography-tandem mass spectrometry (UPLC-MS/MS) metabolite profiling to depict the bile acid spectrum in the serum of CP patients. Controls included healthy controls and the patients diagnosed as other vasculitis (Takayasu’s arteritis) and vascular disease with atherosclerotic lesions (peripheral artery disease). Comparing to healthy controls, the level of CDCA, which is one of the two primary bile acids, and its glycine-conjugated secondary bile acid in human, glycochenodeoxycholic acid (GCDCA), was predominantly elevated in CP patients (**Figure 1**). While deoxycholic acid (DCA), a secondary bile acid converted from the other primary bile acid cholic acid (CA), along with its glycine-conjugated form glycodeoxycholic acid (GDCA), were significantly decreased in CP patients. Of note, the patients of Takayasu’s arteritis showed much more increase in CA, GCA, TCA, TCDCA, DCA, TDCA and tauroursodeoxycholic acid (TUDCA) while the patients of peripheral artery disease showed a dominant increase in CDCA. These results suggest that the bile acid metabolism, especially the metabolism of CDCA and GCDCA, is obviously altered in individuals with CP than normal conditions. The bile acid spectrum of CP is also significantly different from other vascular diseases.

### Altered Gene Sets in CP Patients Are Associated With Bile Acid Metabolism

**Figure 2:**
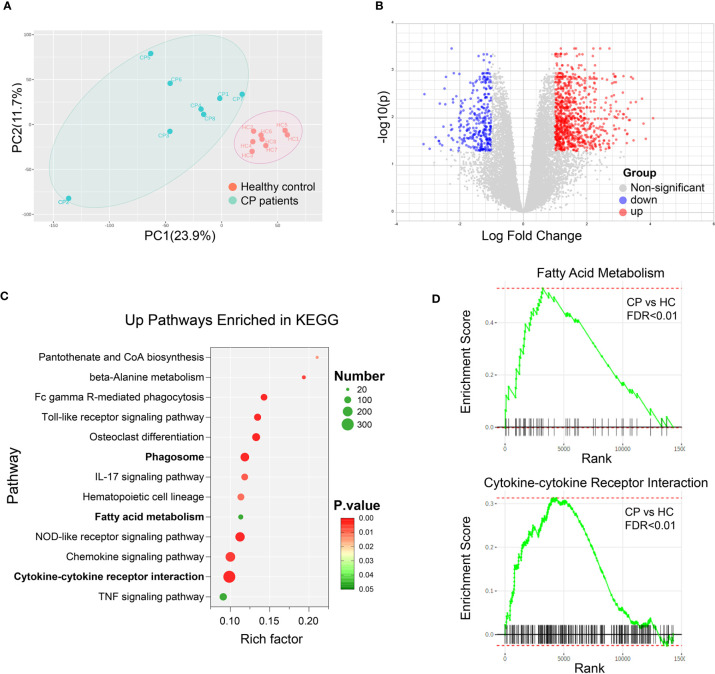
Bile Acid Metabolism is Associated with the Altered Signalings in CP Patients. Peripheral blood mononuclear cells (PBMC) were collected from 8 CP patients along with 8 age-gender matched healthy controls. RNA sequencing and transcript analysis were then performed and gene sets were enriched in KEGG and GSEA. **(A)** Principal component analysis of healthy controls (HC) and CP patients. **(B)** Volcano plot of the differentially expressed transcript analysis in CP comparing to HC. **(C)** Signaling pathways enriched in KEGG with the up-regulated genes in CP compared to HC. **(D)** Representative signaling pathways, fatty acid metabolism and cytokine-cytokine receptor interaction, enriched in GSEA results comparing CP to HC.

In order to investigate the impact of bile acids changes in CP on immune cells, we collected the peripheral blood mononuclear cells from 8 CP patients along with 8 age-gender matched healthy controls and performed RNA sequencing. Principal component analysis showed that the gene sets in CP patients were significantly different from the healthy controls (**Figure 2A**). Differentially expressed transcript analysis revealed that 1336 genes were up-regulated in CP patients compared to healthy controls while 668 genes were down-regulated, as showed in the volcano plot (**Figure 2B**). Based on the differentially expressed genes, Kyoto Encyclopedia of Genes and Genomes (KEGG) pathway analysis was adopted to perform the signaling pathway enrichment. Interestingly, we found that the bile acid metabolism associated signaling pathways (18) were significantly enriched in up-regulated genes, named as phagosome, fatty acid metabolism and cytokine-cytokine receptor interaction (**Figure 2C**). GSEA results also revealed a significant upregulation of the gene sets in fatty acid metabolism as well as cytokine-cytokine receptor interaction (**Figure 2D**). Together, these results suggest that bile acids impact differently on immune cells in CP patients comparing to the healthy controls.

### Pathological Level of CDCA Decreases IL-6 Expression in Macrophages

**Figure 3:**
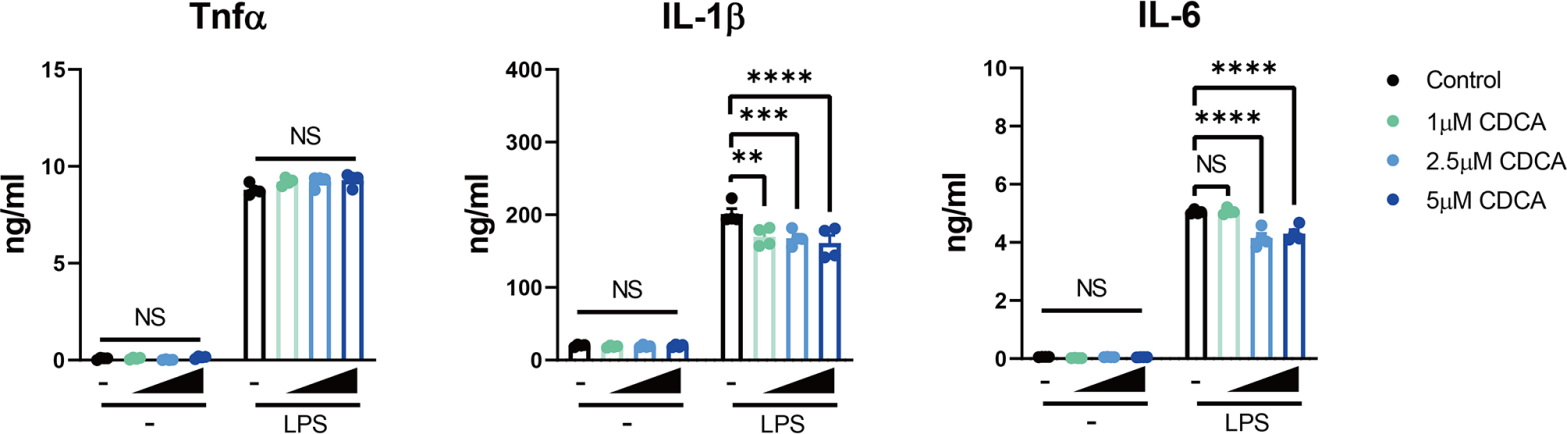
Pathological Level of CDCA Decreases IL-6 Expression in Macrophages. RAW 264.7 macrophages were incubated with or without different concentrations of CDCA for 24h, and then stimulated with or without 100ng/ml LPS. TNFα, IL-1β and IL-6 levels in the supernatant from 24-hr cultures are shown. Data are shown as mean ± SEM. Experiments were repeated 3 times and representative data are shown. **p < 0.01; ***p < 0.001; ****p < 0.0001; NS, no significant difference, two-way ANOVA, followed by Dunnett’s multiple comparisons test.

To investigate the effect of altered bile acids on immune cells, especially the elevation of CDCA, we checked the role of CDCA on macrophages with the murine cell line RAW 264.7. We treated the cells with different concentrations of CDCA for 24h, and then stimulated the cells with 100ng/ml LPS. As expected, we found CDCA at physiological concentration (1μM) did not have the inhibitory role on the expression of pro-inflammatory cytokines TNFα and IL-6, but slightly inhibited IL-1β expression. However, at pathological level (over 2.5μM) as in CP patients, CDCA could significantly decrease IL-6 expression (**Figure 3**).

### FXR Agonist GW4064 Inhibits IL-6 Expression in Macrophages

**Figure 4:**
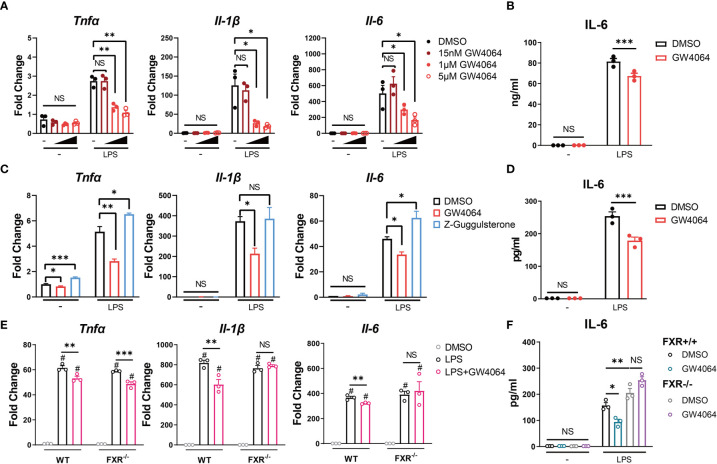
FXR Agonist GW4064 Inhibits IL-6 Expression in Macrophages. **(A, B)** RAW 264.7 macrophages were incubated with or without different concentrations of FXR agonist GW4064 for 24h, and then stimulated with or without 100ng/ml LPS. **(A)** The mRNA levels of TNFα, IL-1β and IL-6 from harvested cells at 6h after the stimulation are shown. **(B)** IL-6 levels in the supernatant from 24-hr cultures are shown. **(C, D)** Bone marrow-derived macrophages were incubated in the presence of DMSO, 5μM GW4064 or 1μM FXR antagonist Z-Guggulsterone for 24h. Cells were then stimulated with or without 100ng/ml LPS. **(C)** The mRNA levels of TNFα, IL-1β and IL-6 from harvested cells at 6h after the stimulation are shown. **(D)** IL-6 levels in the supernatant from 24-hr cultures are shown. **(E, F)** Bone marrow-derived macrophages from FXR knockout (FXR^−/−^) mice and the littermates were incubated with or without 5μM GW4064 for 24h, and then stimulated with or without 100ng/ml LPS. **(E)** The mRNA levels of TNFα, IL-1β and IL-6 from harvested cells at 6h after the stimulation are shown. **(F)** IL-6 levels in the supernatant from 24-hr cultures are shown. Data are shown as mean ± SEM. Experiments were repeated 3 times and representative data are shown. *p < 0.05; **p < 0.01; ***p < 0.001; NS, no significant difference; # means compared to the relative unstimulated control group, two-way ANOVA, followed by Sidak’s multiple comparisons test.

Since CDCA is a well-known natural high-affinity ligand for farnesoid-x-receptor (FXR) (19), we then wondered if the inhibitory role of CDCA on macrophages could be mediated by FXR activation. As GW4064 is the synthetic specific agonist of bile acid receptor FXR, we investigated the impact of GW4064 on function of monocytes/macrophages, especially on the expression of IL-6, the cytokine significantly up-expressed in CD68^+^ macrophages/monocytes of CP patients (16). Firstly, we incubated the RAW 264.7 macrophages with different concentrations of FXR agonist GW4064 for 24h, and then stimulated the cells with 100ng/ml LPS. As showed in **Figure 4A**, the mRNA levels of the pro-inflammatory factors TNFα, IL-1β and IL-6 were significantly up-regulated upon the LPS stimulation; however, GW4064 could significantly inhibit their expressions 6h after the stimulation, in a dose-dependent manner. The protein level of IL-6 in the supernatant of stimulated macrophages was also decreased after the GW4064 treatment (**Figure 4B**). We then consolidate the findings with bone marrow-derived macrophages. We observed a consistent phenotype with a significant decrease of pro-inflammatory factors in both mRNA level and protein level, especially for IL-6 (**Figures 4C, D**). In addition, the incubation of the antagonist of FXR Z-Guggulsterone with BMDM led to an increase of TNFα and IL-6 on mRNA level (**Figure 4C**). Furthermore, FXR knockout did not affect the macrophage differentiation (**Supplementary Figure 1**), however, the incubation of GW4064 with FXR knockout (FXR^−/−^) BMDM revealed no significant inhibition of IL-6 in both mRNA and protein level (**Figures 4E, F**). Taken together, these data indicates that the activation of FXR has a specific inhibitory role on the IL-6 expression, and bile acids, especially CDCA may have an inhibitory role on the IL-6 expression through the activation of FXR.

### FXR Agonist Promotes the Translocation of FXR Into the Nucleus

**Figure 5:**
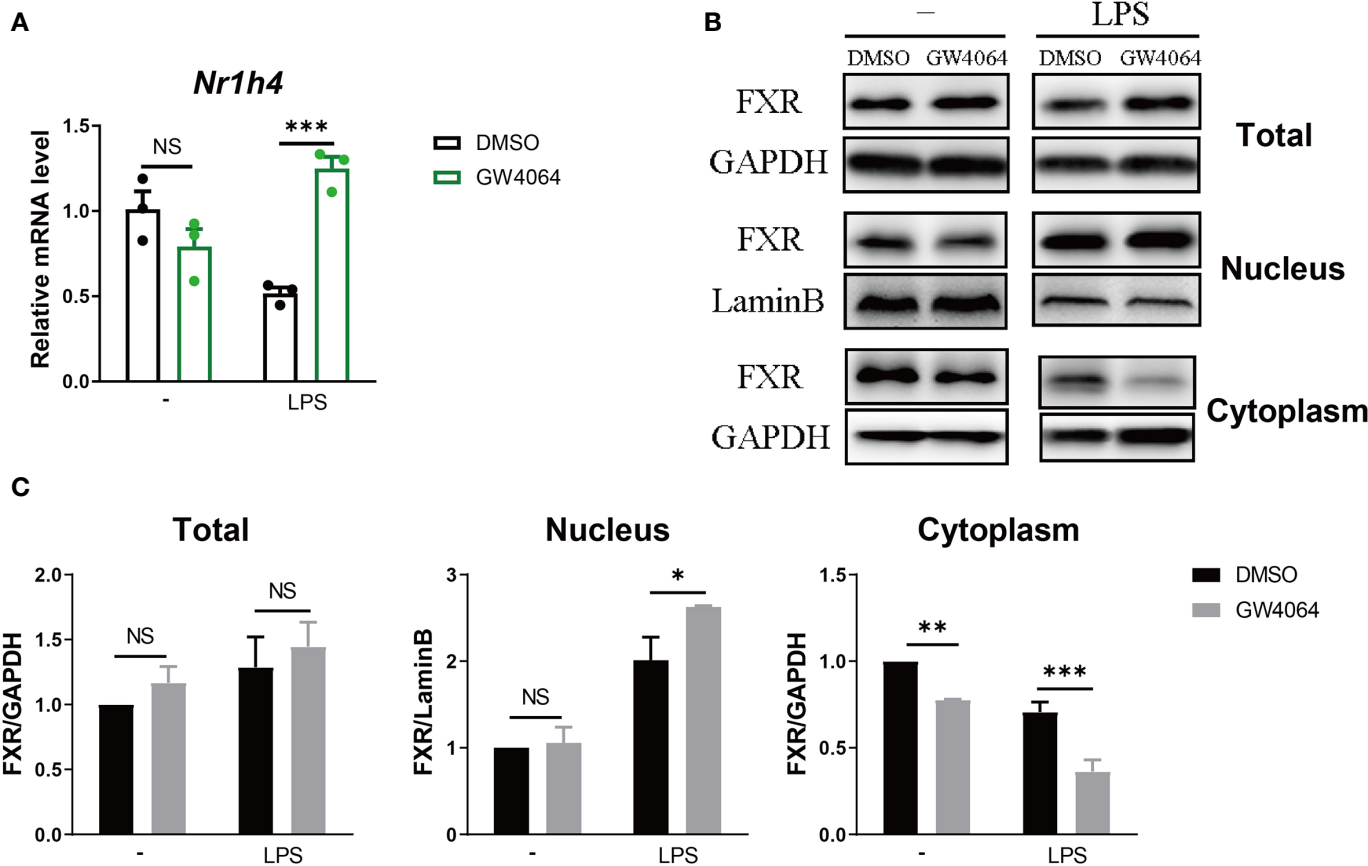
FXR Agonist Facilitates the Translocation of FXR into the Nucleus. **(A, B)** RAW 264.7 macrophages were incubated with or without 5μM FXR agonist GW4064 for 24h, and then stimulated with or without 100ng/ml LPS. The mRNA levels of FXR within the cells **(A)** were tested 6h after the stimulation and total protein levels as well as nuclear and cytoplasmic protein levels were tested 24h **(B)** after the stimulation. **(C)** Quantification of the western results from **(B)**. Data are shown as mean ± SEM. *p < 0.05; **p < 0.01; ***p < 0.001; NS, no significant difference, two-way ANOVA, followed by Sidak’s multiple comparisons test.

We investigated how FXR agonist affects FXR in RAW 264.7 macrophages. We firstly evaluated the FXR expression in the macrophages. Incubation with GW4064 did not significantly change expression of *Nr1h4*, the gene encoding FXR (**Figure 5A**). Under LPS stimulation, cells incubated with GW4064 had significantly increased expression of *Nr1h4* (**Figure 5A**). Protein analysis by western blotting did not reveal a clear increase in total FXR (**Figure 5B**). However, FXR within the nucleus was significantly increased after LPS stimulation. In parallel, cytoplasm expression of FXR was reduced, particularly in GW4064-treated cells (**Figures 5B, C**). These results indicate that LPS stimulation together with GW4064 stimulation of FXR could impact on transcription as well as nuclear translocation of FXR, fitting to its function as a transcription factor.

### FXR Binds to the Promoter Site of IL-6

**Figure 6:**
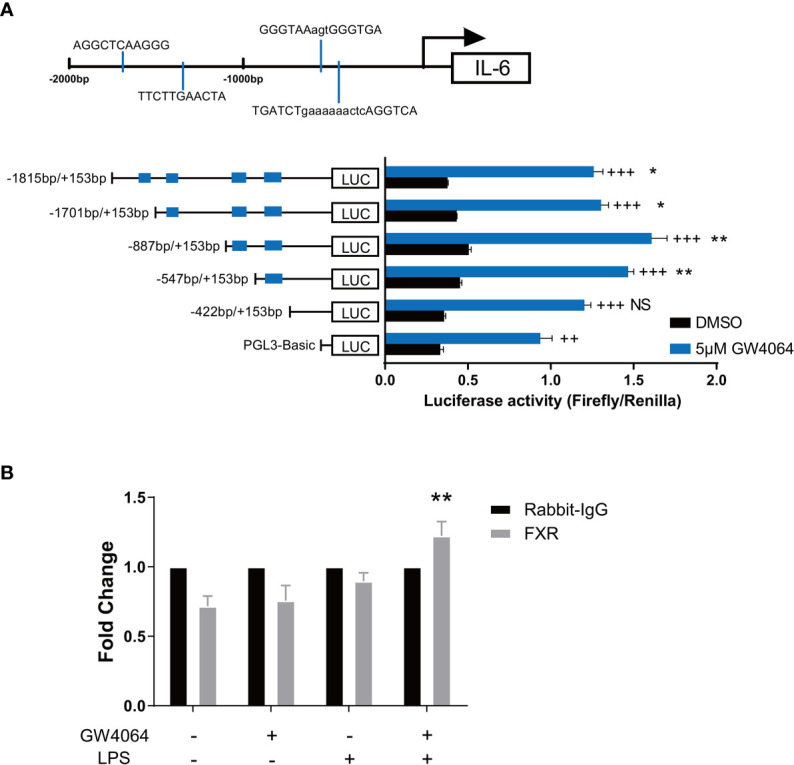
FXR Binds to the Promoter Site of IL-6. **(A)** Four potential FXR binding sites in the promoter site of IL-6 were predicted based on the databases of PROMO and NUBIscan within about 2000bp upstream of the transcription start site of IL-6 (upper). Plasmids containing different numbers of the binding site based on the basic PGL-3 plasmid were constructed and co-transfected respectively with FXR-overexpression plasmids as well as Renilla plasmids together into 293T cells. 24h after the transfection, cells were then activated with or without 5μM GW4064 for extra 24h. Luciferase activity was then evaluated with the dual luciferase assay (bottom). Data are shown as mean ± SEM. + means compared to unstimulated control and * means comparing to the luciferase activity of PGL-3 basic vector. ++ p < 0.01; +++ p < 0.001. *p < 0.05; **p < 0.01; NS, no significant difference, two-way ANOVA, followed by Sidak’s multiple comparisons test. **(B)** RAW 264.7 macrophages were incubated with or without 5μM FXR agonist GW4064 for 24h, and then stimulated with or without 100ng/ml LPS for 24h. Cells were then fixed with 1% w/v formaldehyde solution and chromatin from the cells was analyzed for recruitment of FXR to the last predictive binding site of the IL-6 promoter by ChIP assay. The quantification of DNA in the precipitation with FXR antibody was normalized to input chromatin and plotted relative to the Rabbit IgG. Data are shown as mean ± SEM. **p < 0.01, two-way ANOVA, followed by Sidak’s multiple comparisons test.

We next investigated how GW4064 might regulate IL-6 production by macrophages. Considering that FXR is a transcription factor, we looked into the direct role for FXR in regulating IL-6 transcription. Based on the databases of PROMO and NUBIscan, we performed the binding site prediction of FXR within about 2000bp upstream of the transcription start site of IL-6, and found 4 potential binding sites in the promoter site of IL-6 (**Figure 6A** upper). According to the prediction results, we constructed several plasmids containing different numbers of the binding site based on the basic pGL-3 plasmid (**Figure 6A** bottom, left), performed the co-transfection of them respectively with FXR-overexpression plasmid as well as Renilla plasmid, and tested the luciferase activity with the dual-luciferase assay. The reporter assay showed that comparing to the basic pGL-3 plasmid, FXR could directly bind to the plasmid containing the whole 4 binding sites and significantly promoted the luciferase activity. Moreover, the successive deletion of the former 3 binding sites did not change a lot of the luciferase activity, while the deletion of the last binding site led to a disappear of the significance of the luciferase activity, indicating that the actual binding region of FXR on the promoter of IL-6 is located on the last predictive site (**Figure 6A** bottom, right). The additional ChIP assay was also conducted targeting the last predictive site, and the result depicted that FXR indeed possessed a higher binding activity to the fourth predictive binding site on the promoter of IL-6 under the activation of GW4064 upon LPS stimulation (**Figure 6B**).

### IL-6 and FXR Are Expressed in CP Human Sample

**Figure 7:**
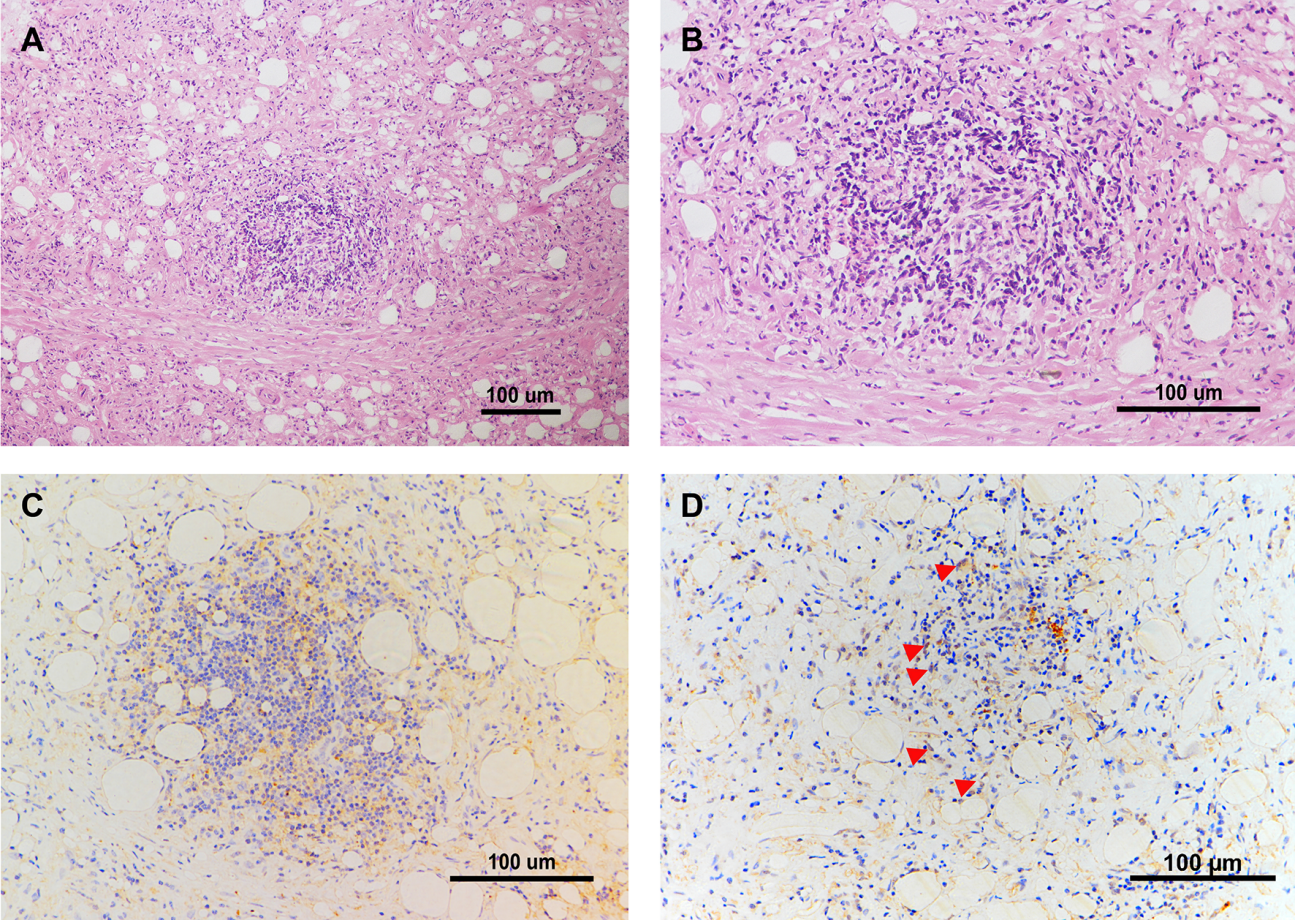
IL-6 and FXR are Expressed in the Retroperitoneal Lesion of CP. **(A, B)** Representative images of HE staining in the biopsy of the retroperitoneal lesion from one CP patient. Scale bars: 100 μm. **(C)** Representative image of immunohistochemical staining of IL-6 in the biopsy of the retroperitoneal lesion. Scale bars: 100 μm. **(D)** Representative image of immunohistochemical staining of FXR in the biopsy of the retroperitoneal lesion. Scale bars: 100 μm. The red arrows indicate FXR positive cells especially within the nucleus.

To further investigate the FXR expression as well as IL-6 expression locally in CP patients, we performed a biopsy of the retroperitoneal lesion from one CP patient and conducted the following HE and immunohistochemical staining. The morphological observation from the HE staining revealed a typical manifestation of CP, with an abundant infiltration of foam-like cells and lymphocytes within the adipose tissues and collagen fibers (**Figures 7A, B**). IL-6 was broadly expressed within the lesion and especially within the site of inflammation (**Figure 7C**). FXR was also expressed within the lesion, especially in the nucleus of the cells (**Figure 7D**). These results suggest that FXR indeed exists in the inflammatory and fibrotic lesion of CP, and may benefit the therapy of CP with its inhibitory role on IL-6 expression in the local site.

## Discussion

In 1905, Albarran first reported three cases of ureteral obstruction caused by extensive retroperitoneal fibrosis. And then in 1972, inflammatory abdominal aortic aneurysm was first reported (20). Later in 1984, Mitchinson found the consistency in the pathogenesis of IRF and IAAA, further putting forward the concept of perianeurysmal retroperitoneal fibrosis (21). Then the three definitions were summarized as the same disease spectrum, collectively referred to as chronic periaortitis.

Incidence of CP is quite rare. No report has been published so far about the overall incidence of CP, while studies has showed the incidence of IRF was 1-1.38/10 million, males more than females, and the average onset age is 50-60 years old (12, 13). CP has an insidious onset and chronic disease progression, often discovered by routine medical examination or serious complications caused by the damage of corresponding organs. Meanwhile, the clinical symptoms as well as conventional laboratory examinations are mostly not specific, contributing to the difficulty with the early diagnosis. Even though the progress of CP is slow, the late diagnosis and even misdiagnose along with the late initiation of the treatment lead to the poor prognosis of the disease. Moreover, no standard treatment has been settled for CP so far and large amounts of glucocorticoid are still considered to be the first choice, with strong side effects and easy to relapse after drug reduction or withdrawal (22, 23). The treatment of conventional disease-modifying anti-rheumatic drugs (DMARDs) such as cyclophosphamide (24), methotrexate (23) is still under exploration, and tamoxifen as well as biological agents such as TNFα antagonists (25), CD20 monoclonal antibodies (26) and anti-IL-6 antibodies (16) are gradually added to backup list, but their effect, however, is still limited. It seems like we are still pale and weak in front of this tough disease.

Therefore, it is worthy looking forward to some novel therapy for this complicated disease. In our present study, we for the first time explored the possibility of FXR agonist in the treatment of CP through the activation of FXR to function as a transcriptional inhibitor for the pro-inflammatory and pro-fibrotic cytokine IL-6, thus not only alleviate the local inflammatory response and also simultaneously ameliorate the fibrosis of retroperitoneal mass, reducing the damage to the surrounding organs. Furthermore, in comparison with healthy controls as with other vasculitis and metabolic vascular disease, the spectrum of bile acids in the serum of CP patients reveals a specific character with a significant increase of CDCA, indicating a spontaneous defense reaction of the body to inhibit the progression of this disease. Meanwhile, the signaling enrichment analysis of the transcripts also suggests the participation of bile acid metabolism in CP. Thus, increased CDCA may be a new biomarker of this disease, and CDCA supplementation or CDCA analogue as with FXR agonist may be a novel method in the treatment of CP. Recently, the rapid development of new FXR agonists such as obeticholic acid (9) have gained good results from a series of clinical studies, providing a bright future for the treatment of liver fibrosis, and new efficient FXR agonists could also make up for the liver toxicity of CDCA. Expansion of these drugs from the utility only in liver and intestine disease to CP could further provide perspective and promising treatment for CP, and the future randomized clinical trial is worthy to be considerate.

It is reported that level of serum IL-6 was significantly elevated in CP patients than the healthy controls (*p*<0.05) (16). Our immunohistochemical staining results also showed that IL-6 was highly expressed in the retroperitoneal mass of active CP patient, which indicates that IL-6 might be a vital cytokine involved in the inflammatory and pro-fibrotic response of CP. Moreover, although mainly expressed in liver and intestine, FXR was also observed for the first time existing in the retroperitoneal mass, and may function locally in the transcriptional regulation of IL-6. CDCA-FXR/IL-6 pathway may benefit for the resolution of CP by enhancing the inhibitory effect of FXR on IL-6.

However, there are still several problems worthy of improvement in this study. Due to the rare incidence of the disease, few CP patients were recruited and the data was still limited, thus the sample size should be further expanded in the future; serum CDCA level can be influenced by many factors such as diet, drugs, and diseases like hypertension, diabetes and other metabolic diseases. In addition, urinary system is also involved in the metabolism of CDCA, whether the invasion of the retroperitoneal mass into the urinary system contributes to the accumulation of CDCA remains undetermined. In future studies, these confounding factors should be focused and controlled as much as possible. Furthermore, the mechanism underlying the spontaneous increase of CDCA in CP patients remains unknown. Since the metabolism of bile acids is often connected with microbiota (27), the alteration of the microbiota within the gut of CP patients remains to be further elucidated. This aspect seems quite interesting and warrants further investigation. What’s more, until now there is still no appropriate animal model for the investigation of CP, which is also the direction we should figure out in the future.

In conclusion, our results suggest a reactive increase of CDCA in CP patients, which could help to downregulate the pro-inflammatory and pro-fibrotic factor IL-6 in macrophages by activating the transcription factor FXR and then directly binds to the promoter site of IL-6. Thus this could be a promising research direction and treatment strategy for CP in the future.

## Data Availability Statement

The original contributions presented in the study are included in the article/**Supplementary Materials**. Further inquiries can be directed to the corresponding authors.

## Ethics Statement

The studies involving human participants were reviewed and approved by Ethics Committee of Renji Hospital. The patients/participants provided their written informed consent to participate in this study. The animal study was reviewed and approved by Ethics Committee of Renji Hospital. Written informed consent was obtained from the owners for the participation of their animals in this study. Written informed consent was obtained from the individual(s) for the publication of any potentially identifiable images or data included in this article.

## Author Contributions

SC and XC designed the study and wrote the manuscript. XM contributed to network reconstruction. SC performed sequencing analysis. SC performed *in vitro* experiments and analysis. XM and YL analyzed human samples. LS, LJ, HX, and XC collected human samples. XC supervised the study and edited the manuscript. All authors contributed to the article and approved the submitted version.

## Funding

This study was supported by the National Science Foundation of China (81771729, 81971534).

## Conflict of Interest

The authors declare that the research was conducted in the absence of any commercial or financial relationships that could be construed as a potential conflict of interest.

## Supplementary Material

Supplementary Figure 1 FXR Knockout does not Affect the Macrophage Differentiation. On Day7 of the differentiation, the BMDM from FXR^-/-^ mice and the littermates were harvested and stained. The percentage of the differentiated macrophages (CD11b^+^ F4/80^+^), M1 macrophages (CD11b^+^ F4/80^+^ CD11c^+^ CD206^-^) and M2 macrophages (CD11b^+^ F4/80^+^ CD11c^-^ CD206^+^) were then calculated. **(A)** Representative images of the gating strategy for the macrophages in two groups. **(B)** Percentage of the macrophages in two groups. Data are shown as mean ± SEM, NS means no significant difference, student t-test.

## Abbreviations

CP, chronic periaortitis; FXR, farnesoid-X-receptor; IRF, idiopathic retroperitoneal fibrosis; IAAA, inflammatory abdominal aortic aneurysms; PARF, perianeurysmal retroperitoneal fibrosis; BMDM, bone marrow-derived macrophages; PBMC, peripheral blood mononuclear cell; TA, Takayasu’s arteritis; PAD, peripheral arterial disease; UPLC-MS/MS, ultra performance liquid chromatography-tandem mass spectrometry; DEG, differentially expressed gene; KEGG, Kyoto encyclopedia of genes and genomes; GSEA, gene set enrichment analysis; DMARDs, disease-modifying anti-rheumatic drugs; CA, cholic acid; TCA, taurocholic acid; GCA, glycocholic acid; CDCA, chenodeoxycholic acid; TCDCA, taurochenodeoxycholic acid; GCDCA, glycochenodeoxycholic acid; DCA, deoxycholic acid; TDCA, taurodeoxycholic acid; GDCA, glycodeoxycholic acid; LCA, lithocholic acid; TLCA, taurolithocholic acid; GLCA, glycolithocholic acid; UDCA, ursodeoxycholic acid; TUDCA, tauroursodeoxycholic acid; GUDCA, glycoursodeoxycholic acid.
