# Supplementary figures and images for: Bile Acids Elevated in Chronic Periaortitis Could Activate Farnesoid-X-Receptor to Suppress IL-6 Production by Macrophages
Source: Front Immunol. 2021 Apr 22;12:632864. doi: 10.3389/fimmu.2021.632864 (PMC8100322; doi:10.3389/fimmu.2021.632864)

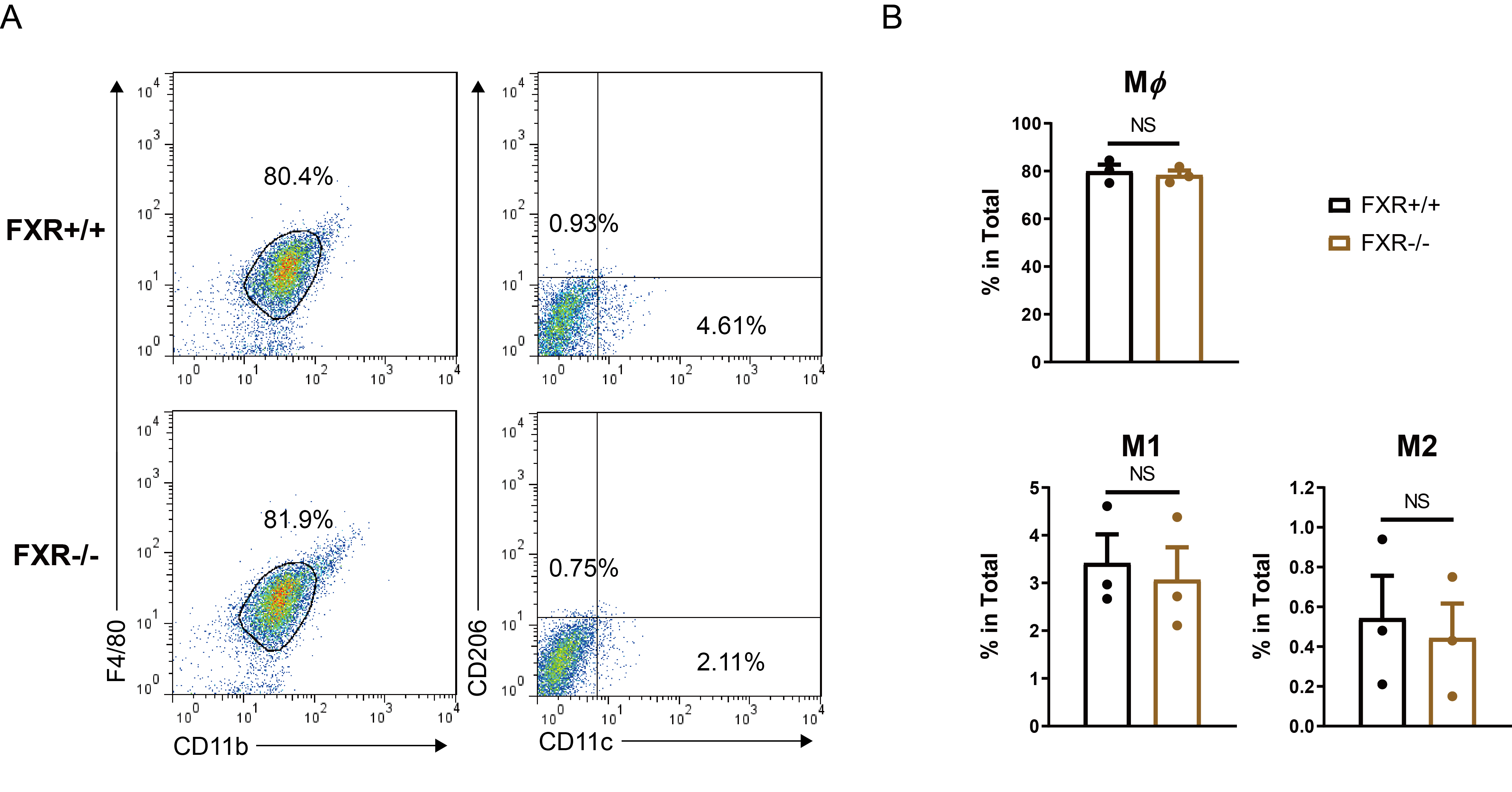

Supplement: Supplementary Figure 1 — FXR Knockout does not Affect the Macrophage Differentiation. On Day7 of the differentiation, the BMDM from FXR-/- mice and the littermates were harvested and stained. The percentage of the differentiated macrophages (CD11b+ F4/80+), M1 macrophages (CD11b+ F4/80+ CD11c+ CD206-) and M2 macrophages (CD11b+ F4/80+ CD11c- CD206+) were then calculated. (A) Representative images of the gating strategy for the macrophages in two groups. (B) Percentage of the macrophages in two groups. Data are shown as mean ± SEM, NS means no significant difference, student t-test. [file Image_1.jpeg]
